# Exploring the Potential for Tuition-Free Higher Education in South Africa: A Scoping Review

**DOI:** 10.12688/f1000research.150265.1

**Published:** 2024-05-22

**Authors:** Tlotlo Ramasu, Grace Kanakana-Katumba

**Affiliations:** 1 Industrial Engineering, Tshwane University of Technology, Pretoria, Gauteng, South Africa

**Keywords:** Free education, Gross Domestic Product, Household Income, Higher Education, South Africa.

## Abstract

The debate on tuition-free higher education has raged in South Africa since the #FeesMustFall protests at South African universities in 2015 and 2016. The government responded with a tuition-free education policy targeting students from households earning less than R350 000. However, the question remains can South Africa sustain a tuition-free education policy given its developing nation status and the levels of its GDP? This article sought to assess the feasibility of tuition-free higher education for all in South Africa. A scoping review was used, and fifteen articles about tuition-free higher education feasibility in South Africa were reviewed. The choice of the scoping review was due to the need for an understanding of the current state of play in research on the feasibility of tuition-free higher education in South Africa. The results suggest that tuition-free higher education for all is not feasible in South Africa. There seems to be a consensus that South Africa lacks the resources to finance tuition-free higher education for all. Tuition-free higher education is also viewed as a regressive tax on the poor given that the majority of students in higher education institutions come from middle and upper-income households. However, it is important to note that a distinction is drawn between tuition-free higher education for all and tuition-free higher education for the poor and academically deserving. The latter seems to receive support with some viewing it as a moral imperative in an unequal society such as South Africa’s. It is recommended that future studies approach the issue from an empirical standpoint whereby the GDP levels are assessed against higher education funding.

## Introduction

For most students, the monetary costs of attending university, college and other higher education institutions are becoming prohibitive. In South Africa, the average cost of higher education is R55 900 per year ( Mafilika, 2023), while the average annual gross salary in South Africa is R303 648 ( Davis, 2023, Statistics South Africa ( StasSA, 2023a, b). Housing and living costs add an extra burden on students. The result is that those from poor households and historically disadvantaged communities cannot afford to attend university ( Chiramba & Ndofirepi, 2023). Some countries have resorted to offering loans to students to make it easier for them to afford education. However, this has resulted in students being saddled with high levels of debt, which tend to adversely impact other aspects of life, such as family formation and home ownership ( Beal et al., 2019; Goldrick-Rab & Steinbaum, 2020; Velez et al., 2019).

To ensure access to education for all, some countries have instituted policies that ensure education is free for all citizens and in some cases for international students ( de Gayardon, 2019). The majority of countries with free education systems up to higher education levels are in the developed world such as Austria, Belgium, Brazil, Cyprus, Czech Republic, Denmark, Egypt, Finland, France, Germany, Greece, Hungary, Iceland, Italy, Luxembourg, Malta, Norway, Panama, Poland, Saudi Arabia, Sweden, Spain, Slovenia, Switzerland, Slovakia, Turkey and Uruguay ( Chang, 2020; Erudera, 2023). In these countries free education is publicly funded, meaning taxes are the main source of funding ( Hügle, 2021).

In 2015, South Africa experienced mass protests by students under the banner of #FeesMustFall ( Ntombana et al., 2023). The protests started after the University of the Witwatersrand proposed a fee increase for the 2016 academic year. This was followed by protests in all government-funded universities. These protests birthed the demand for free higher education. This was in recognition of the fact that the South African education system was failing to cater for the needs of the historically marginalized and oppressed. On 16 December 2017 President Jacob Zuma surprised the country by announcing the introduction of tuition-free education at the higher education level. The announcement indicated that those in households with a combined income of less than R350 000 would be funded by the government ( Department of Higher Education and Training (DHET), 2018). While the announcement was greeted with jubilation among the poor and working class, some experts raised the issue of the capability of the government to fund the system ( Hlaka & Sefoka, 2022; Mdepa, 2022). Scepticism of the success of the new policy also arose due to the failures experienced with the National Student Financial Aid Scheme (NSFAS) loan system. The NSFAS loan system was designed to provide assistance to impoverished students. However, the loan faced challenges with some deserving students failing to access the loan and with low repayment rates ( Ntombana et al., 2023).

Education plays an important role in national development ( Kioupi & Voulvoulis, 2019). As a developing nation, South Africa is in need of an educated workforce. South Africa is faced with high levels of income inequality with a Gini coefficient of 0.67 which is the highest in the world ( Friderichs, 2021). Education can play a role in bridging the gap between the wealthy and the poor. South Africa also faces the challenge of a high unemployment rate with a recorded 31.9% unemployment rate (StatsSA, 2023b). With many of the unemployed lacking skills, education can play a role in improving their employability. The introduction of free higher education for the poor was done with the belief that education would bring about equality as those formally marginalized would be able to participate in higher education resulting in gainful employment that would improve their economic conditions ( DHET, 2018). However, South Africa is still a developing country and faces a myriad of challenges which demand the attention and resources of the government thus free education might impact other government programs.

South Africa’s GDP is currently recorded at US$405 billion dollars, or US$6766 per capita ( World Bank, 2022a). In comparison to other countries in the developed world offering free education, South Africa has low levels of GDP per capita. For example, Denmark has a GDP of US$ 400 billion, US$67 790 per capita ( World Bank, 2022b). The average GDP per capita in Europe, a significant proportion of European countries that offer free education, is above US$37 430 ( World Bank, 2023). The different economic realities those in the developed world find themselves and those faced by South Africa raise the question of whether South Africa is suited and prepared for free education in the long term. The issue of tuition-free higher education has been emotive with both sides, proponents and opposers, coming up with arguments that seem legitimate. To get a clear understanding of the issue a review of literature is warranted. Thus the is a need for a scoping review to have a clear understanding of what research studies indicate on the subject. The article seeks to assess if South Africa is able to sustain free education funding in the long term given the level of its GDP.

## Literature review

### Reasons for free education

***Higher education as a public good?***

A major point of contention between those who are pro-free education and those against has been whether education is a private good or public good ( Tilak, 2008; Williams, 2016). Those who argue that education is a private good point to the many benefits that accrue to individuals due to being educated, while those who argue it is a public good consider education as key to society’s betterment and thus serve the public interest ( Marginson, 2007). In Nordic countries where an economic and social model has been developed towards the upliftment of society as a whole, education is considered a public good thus they have adopted tuition-free education ( Antikainen, 2016). Public goods are described as non-rivalrous and non-excludable ( Marginson, 2011; Samuelson, 1954). Consumption of non-rivalrous goods does not lead to their exhaustion while non-excludable goods have their benefits extending beyond the consumer ( Williams, 2016). It has been argued that education does not fully meet the characteristics of non-rivalry and non-excludability ( Marginson, 2018). Entry into top higher education can be rivalrous due to limited spaces and the benefits of education do accrue to individuals making them excludable ( Nixon, 2017). However, education has been noted to have benefits for society as a whole thus investment in education can be for the “public good” ( Marginson, 2018). South Africa’s cost-sharing model whereby the government contribute a share and individuals contribute a share as well is borne out of the idea that higher education generates both private good and public good ( De Jager & Baard, 2019).

***Higher education is a right?***

Proponents of tuition-free higher education argue that education is a right thus the state has a role in ensuring that those who wish to be educated are accommodated ( Burke, 2013; Gilchrist, 2018). This points to the state working towards removing all obstacles for those who want to participate in higher education. For some students, tuition fees can be an obstacle. Education can be viewed as a social right that should be afforded to every citizen for the betterment of society as a whole ( Lee, 2013).

***Promotion of equality***

In a world increasingly marked by inequality, with the rich increasingly becoming richer while the poor become poorer, education is regarded as a promoter of equality ( Li et al., 2022). Proponents of free education call for equality in access to higher education as a cure to economic, and social inequality ( Dias, 2015; Sá & Serpa, 2022). Thus the call for all students to have equal opportunities in accessing education despite their social and economic background, religion, gender, race, and other differentiating factors. Education is considered to be human capital and studies have indicated that improving the human capital of a nation can lead to economic growth ( Gyimah-Brempong et al., 2006). Improvements in a nation’s human capital can lead to improved productivity and better technologies ( Abel et al., 2012; Baharin et al., 2020). Education also has other benefits for the public such as better health, improved workforce flexibility, increased government tax revenues and higher consumption ( Bloom et al., 2006; de Gayardon, 2019; Vossensteyn, 2009).

***Higher education in South Africa***

It would not be proper to discuss South Africa’s education systems without mentioning the Apartheid Era. It can be argued that some of the inequalities in the current education system are a result of Apartheid-era policies that discriminated against large sections of the population ( Mekoa, 2018). The advent of democracy in 1994 brought about a new government which focused on making education accessible for the population as a whole ( Mzangwa, 2018).

Funding for public higher education institutions in South Africa flows from three main sources; government grants, student tuition and other fees, and other private income which can be in the form of donations ( Steyn & De Villiers, 2007; Wangenge-Ouma & Cloete, 2008). According to Khuluvhe and Netshifhefhe (2021), government grants constitute 42% of the source of funds for public higher education, while student tuition and other fees contribute 33%, and other private income contributes 25% each. This indicates the massive role the government has in the funding of public higher education institutions.

In comparison to other developing countries South Africa’s spending on education is not particularly remarkable; the government spends around 0.9% of GDP ( Yende and Mthombeni, 2023), while other developing countries such as Brazil spend 1.1% of GDP and Chile spends 1.26% of GDP ( DHET, 2018). In recent years the South African higher education sector has seen a decrease in government subsidies thus the burden on students has increased ( De Jager & Baard, 2019). The portion of higher education institutions’ income coming from students rose from 24% in 2000 to 35% in 2015 ( DHET, 2018). It is important to note that the student portion of money spent on higher education has some of it originating from government coffers, for example in 2015 about 40% of the fees paid by students were from NSFAS ( DHET, 2018).

NSFAS has been one of the main vehicles to directly fund individual students who lack the funds to attend University. NSFAS funding has seen a more than fivefold increase from R5.9 billion in 2014 to R34.7 billion in 2020 ( BusinessTech, 2021). On 16, December 2017 President Jacob Zuma announced the introduction of tuition-free education at higher education level for some students. The announcement indicated that those in households with a combined income of less R350 000 would be funded by the government ( Department of Higher Education and Training (DHET), 2018). The announcement indicated that NSFAS would now be granting bursaries rather than loans ( Mavunga, 2019). This gave rise to the question; is South Africa ready for tuition-free higher education? This article sought to assess the feasibility of tuition-free higher education for all in South Africa given its level of GDP based on literature.

### The evolution of South African policies on higher education

Policy initiatives and government actions under the Apartheid government resulted in 19 racially and ethnically composed education departments ( Bunting, 2006). Under the National Party apartheid government, higher education institutions had to have clear designations of which race group they were serving ( Beale, 1998). Universities designated for whites received the bulk of funding and were more resourced than universities for other racial groups ( Beale, 1992; Bunting, 2006). The participation rates in higher education reflected the inequality of the apartheid policies with whites having a 70% participation rate while they had only 10% of the population, blacks it was 9% while they constituted 80% of the population, coloureds it was 13% and for Indians it was 40% ( Sehoole & Adeyemo, 2016).

The post-Apartheid administrations of South Africa have worked to reverse the policies of the Apartheid State ( Wangenge-Ouma, 2012). Several policies have been promulgated to this effect ( Mzangwa, 2019). These policies have been designed to ensure equal access to higher education and to ensure the affordability of higher education for the formally marginalized and the poor ( Akala, 2023). Among them are Education White Paper 3, the National Student Financial Aid Scheme Act, 1999 (Act No. 56 of 1999, and The National Plan for Higher Education ( Akala, 2023; Sehoole & Adeyemo, 2016).

Education White Paper 3 was promulgated in 1997 with one of its main goals being the broadening of access and participation in higher education irrespective of race, gender, ability or age ( Boughey & McKenna, 2021). The policy also sought to redress the effects of Apartheid policies and to eradicate discrimination ( Dreyer et al., 2020). With increased participation of historically marginalised groups, the policy is viewed as having been successful to a certain extent ( Akala, 2023). However, groups such as students with disabilities are still facing challenges in participating in higher education ( McKinney & Swartz, 2022).

The NSFAS Act (Act No. 56 of 1999) provided for the creation of a loan and bursary scheme that catered for eligible students coming from low-income households ( Ede et al., 2022; Pillay et al., 2021). The loan and bursary scheme loan as NSFAS has played a critical role in facilitating the participation of formerly marginalised groups and of those from low-income households ( de Villiers, 2023). As of 2017, the loan scheme has been transformed into a bursary scheme to cater for students from households earning less than R350 000 ( DHET, 2018).

The National Plan for Higher Education was promulgated in 2001 with the goal of providing an implantation framework for policies such as Education White Paper 3 ( Akala, 2023). The policy sought to ensure that the right to education for all became a reality in South Africa ( Mouton, Louw & Strydom, 2013).

Funding was one of the most important mechanisms used by the Apartheid government to ensure to achievement of set policy goals ( Wangenge-Ouma, 2012). The post-Apartheid government of South Africa has recognised that to reverse the negative effects of Apartheid policies there is a need to make use of funding as a tool.

## Method

The study used a scoping review to review literature on tuition-free higher education. A scoping review “is a preliminary assessment of the available literature to identify the nature and extent of the evidence (usually including ongoing research)” ( Tulandi & Suarthana, 2021). To ensure scoping reviews are rigourous, methodological and transparent, the Preferred Reporting Items for Systematic Review and Meta-Analysis Extension for Scoping Reviews checklist was designed ( Tricco et al., 2018). The guidelines provided by the (PRISMA) Extension for Scoping Reviews statement were used to guide the review. The protocol for the review was registered and the details are on the ‘Data availability’ Extended data section.

### Inclusion and exclusion criteria

The inclusion criteria called for articles published in English, articles that focused specifically on tuition-free education, higher education and South Africa. Articles which focused on tuition-free education at other levels of education such as primary and secondary education were excluded from the study. The articles considered also included grey literature that discussed tuition-free higher education in South Africa. Studies using quantitative, qualitative and mixed method methodologies were included to give a broad view of the subject.

### Information sources

Articles were sourced from Google Scholar and Scopus. The search sought articles between the years 2015 and November 2023. However, articles outside of these dates were used when found through the snowball method and when considered relevant. The snowball method was also used to locate articles not identified through the search parameters.

### Search strategy

#### Keywords used in the search for articles include (“tuition”) AND ("free") AND ("tuition") AND ("higher education"), AND ("South Africa"). The search only focused on studies undertaken in English. The search for grey literature was done by inputting the keywords in the Google search engine.

### Selection process

The researcher screened and selected the articles on her own. Each report was screened through reading the title, abstract and conclusion. The search for articles yielded 125 articles. After removing duplicates, 120 articles were left. After reading through the selected articles, 3 articles were identified from the references of the articles. These articles were added to the final list of articles used in the study. Three articles seemed to meet the inclusion criteria but were found to be focused on tuition-free education in primary and secondary education and thus were excluded.

The result of screening the articles was 15 articles published between the years 2008 to 2022. One article looking at free education in other countries was included. This was done when the article were viewed to provide insights that might be helpful in a South African context. Identified articles were downloaded from the databases. Analysis of the articles entailed first examining the abstract, keywords, and methodology used in the study. This was followed by reading the whole article, in some cases reading several times to gain a deep insight into the conclusions reached.

**Figure 1.**
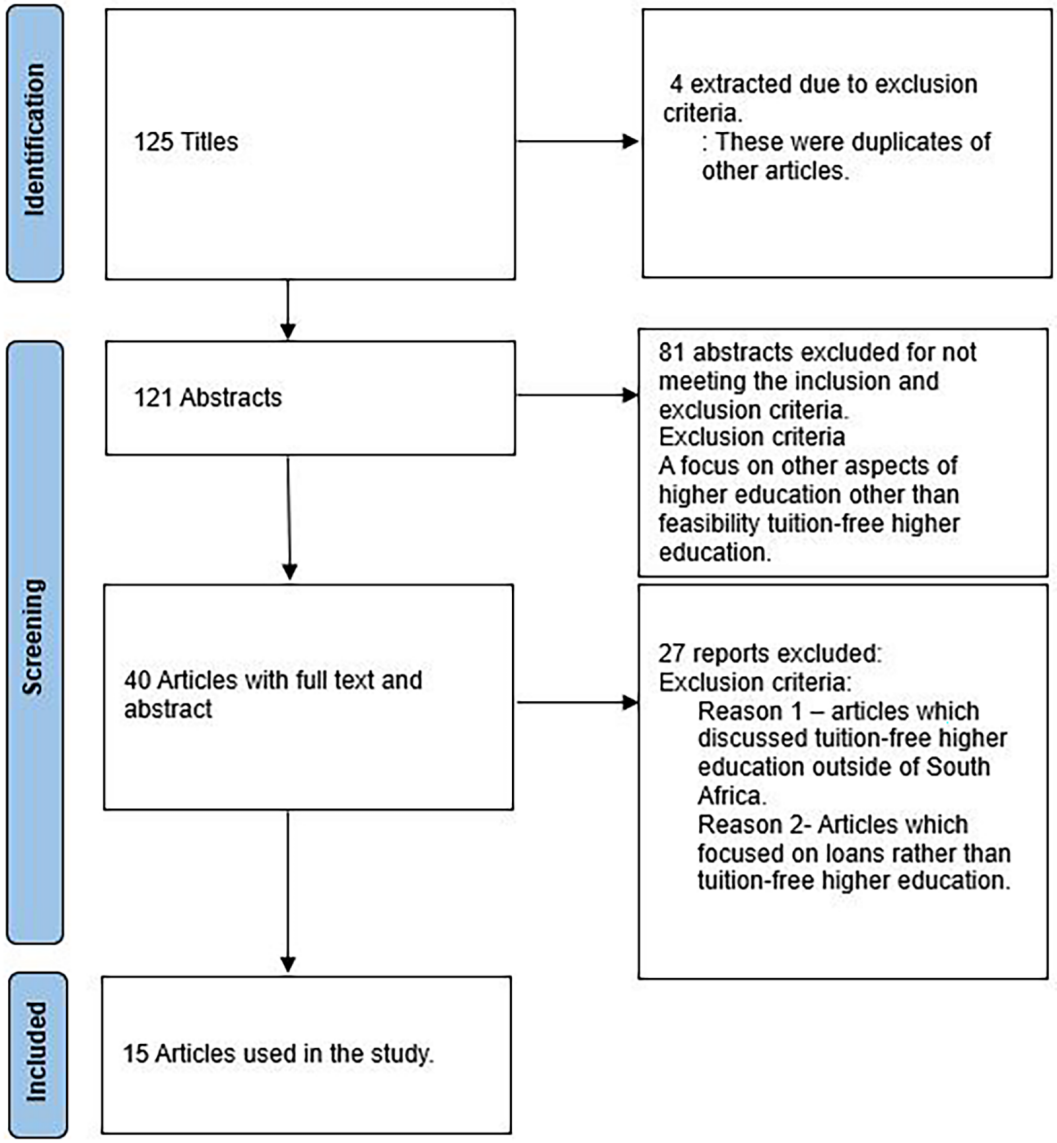
Study selection.

The inclusion and exclusion criteria called for studies undertaken in South Africa, focused on tuition-free higher education and undertaken in the English language. Figure 1 shows the selection procedure for the articles.

### Charting the data

Data charting was undertaken by one researcher. The data charting consisted of identifying the year of publication, the methodology used in the studies and information on tuition-free higher education. Focusing on the issue of tuition-free higher education the content of the studies was analysed. The focus was on the arguments for and against tuition-free higher education and the reasons offered in the studies ( Figure 1).

## Results

### Study characteristics

Of the 15 articles used in the study, one (7%) was published between the years 2005 and 2010, two (13%) between 2010 and 2015, 11 (73%) between 2016 and 2020, one (7%) between 2020 and 2023. The majority of the articles being published between 2016 and 2020 might be due to it being the period where the debate of tuition free education was at its peak due to the #feesmustfall protests that began in 2015. All but one (7%) articles were published in South Africa, the one which focused on other countries was included as it offered insights deemed useful to the South African context. Of the articles used in the study, six (40%) were grey literature and nine (60%) were academic articles. Of the nine academic articles, eight were based on qualitative designs, while one used quantitative designs. Table 1 and Table 2 provide a summary of the characteristics of the articles used in the scoping review.

**Table 1.** Characteristics of sources of evidence.

Characteristic	Categories for each characteristic	Number of studies (n=15)
**Date of publication**	2005-2010	1
	2010-2015	2
	2016-2020	11
	2020-2023	1
**Place of publication**	South Africa	14
	Outside of South Africa	1
**Type of Study**	Academic (Qualitative design)	8
	Academic (Quantitative design)	1
	Grey literature	6

**Table 2.** Study characteristics.

Author (Year)	Study design	Research question/Research objective	Country
Badat (2011)	Grey Literature	Is free higher education necessarily an undesirable ideal?	South Africa
Cloete (2015)	Grey literature	Is free higher education a good idea, and where will the money come from?	South Africa
de Gayardon (2019)	Qualitative study	To explore the philosophical rationales behind the idea of free tuition in countries implementing free education.	Outside South Africa
de Jager and Baard (2019)	Qualitative design	Does “free” higher education in South Africa make economic sense?	South Africa
DHET (2018)	Grey Literature	To Identify investment trends in post-school education and training in South Africa.	South Africa
Dunga and Mncayi (2016)	Quantitative design	Should higher education be free or not in South Africa?	South Africa
Hlaka and Sefoka (2022)	Qualitative design	Can free education be realised in South Africa?	South Africa
Langa et al. (2017)	Grey literature	Is free higher education an illusion?	South Africa
Mlambo et al. (2017)	Qualitative design	The study explores whether the provision of free higher education in South Africa is a proper concept or a parable.	South Africa
Motala et al. (2018)	Qualitative design	What might be included in any discussion about the costs of education.	South Africa
Public Servants Association (2016)	Grey literature	To analyse the challenges facing higher education in South Africa.	South Africa
Sefoka (2018)	Qualitative design	To evaluate the possibility of the South African economy having to fund or cater for free and sustainable higher education.	South Africa
Universities South Africa (2017)	Grey literature	Universities Funding in South Africa; A Fact Sheet.	South Africa
Wangenge-Ouma and Cloete (2008)	Qualitative design	How are universities funded in South Africa?	South Africa
Yende and Mthombeni (2023)	Qualitative design	To discuss unforeseen financial constraints in South African higher education due to fee-free education.	South Africa

## Discussion

This scoping review sought to assess the ability of the South African government to meet the resource demands of free education. Wangenge-Ouma and Cloete (2008) contend that free education is too costly for South Africa and that difficult trade-offs need to be made to fund it as the government has finite resources which are needed in many sectors of the economy. Those arguing against free higher education in South Africa point to the fact that most of the students eligible for higher education come from high-income families thus funding free higher education will be essentially a tax on the poor ( Cloete, 2015; Wangenge-Ouma & Cloete, 2008).

In January 2016, the South African government established the Commission of Inquiry into Higher Education and Training (also known as the Heher Commission) to investigate the feasibility of making higher education and training free ( DHET, 2018). The interim report realised by the Heher Commission in November 2016 recommended that the government fully fund students from households with less than R122 000 annual income and also provide funding for the ‘missing middle’ students from households exceeding the upper limit (R122 000) but in need of funding. The final report deemed tuition-free education to be not feasible for South Africa and suggested a funding model whereby students are funded through income-contingent loans rather than the NSFAS loan. The income-contingent loans would be offered by banks and students would pay them back upon being employed. The Heher Commission viewed the NSFAS loan system to be plagued by numerous problems and inefficiencies hindering its success. The Heher Commission proposed that TVET colleges be free and that they be improved upon so they could be first-choice institutions for some students ( DHET, 2018).

Cloete (2015) argues that free education in a developing country of the ilk of South Africa is “financially, empirically and morally wrong” (p.11). The author calls for the conversation to turn from free higher education for all to affordable higher education for all. This would mean students from different income groups would have to pay different costs. The author feels that it is morally indefensible to require the poor who earn amounts below a certain threshold to pay for higher education. In the same vein, the author points to it being morally indefensible for the rich not to pay anything for higher education. The author warns that if the government does not address issues related to the missing middle, that is the group which does not qualify for NSFAS yet finds it difficult to get loans, protests in the form of the Arab Springs will arise or a more scary revolution in the form of the French Revolution might occur.

Langa, Wangenge-Ouma, Jungblut and Cloete (2017) point to past attempts at instituting free education in some African countries, among them Kenya, Mozambique and Uganda. The tuition-free higher education regime in these countries resulted in increased inequalities between the rich and the poor ( Cloete, 2015). This was due to children of the political and business elites getting access to free higher education at the expense of the deserving and academically gifted poor. In addition, the attempts in these African countries to implement free higher education proved very costly in the long run resulting in poor public higher education institutions. Langa, Wangenge-Ouma, Jungblut and Cloete (2017) argue that free education in a highly unequal society, such as South Africa, can result in higher education benefits only accruing to the already privileged who already have the “economic, social and cultural capital necessary to participate and succeed in higher education”.

de Gayardon (2019) assessed free higher education systems around the world. The author argues that free higher education does not naturally lead to increased access and success in higher education. The author points out that tuition-free higher education systems around the world are diverse economically and geographically. In countries with free education, philosophical and historical traditions drive the adoption of the free higher education policy. In some countries, free higher education is viewed as a right while some view it as a way to promote equal opportunities. The author points out that in some countries that offer free higher education, adjustments have had to be made to systems to ensure financial stability. In some cases this includes having a dual track system where some get free higher education while others pay, in some cases there has been an increased reliance on private institutions which require fees while public institutions remain free.

Badat (2011) argues that the likely consequence of free education for all is that public higher education institutions will lose much-needed resources leading to poor outcomes, which may necessitate a movement of the wealthy to private institutions or overseas. Need for access to real opportunities for success. The author posits the idea of graduate tax whereby beneficiaries of government funding pay a tax, the revenue which is used to fund other students. Badat (2011) makes a distinction between free education for all and free education for those in need, advocating for the latter as important for social and economic mobility.

Yende and Mthombeni (2023) analysed the effect of tuition-free higher education. Their study used qualitative content analysis to analyse documents linked to tuition-free higher education. They looked at the issue through the lens of supply and demand as explained in economic theory. The authors pointed out that the current 0.9% of GDP spent on higher education was deemed insufficient and the South African government needed to double this amount to cover tuition-free education. They also posited that the government needs to increase GDP by at least R2.88 trillion to sustainably implement tuition-free higher education. The authors noted that money for tuition-free education was sourced from tax resources and that currently South African taxpayers were overburdened. The authors point out that one of the consequences of tuition-free higher education for the poor has been a shift from the economic burden from students and parents to higher education institutions. With increased enrolment due to tuition-free education, institutions face the burden of managing dwindling resources with a larger group of students. Dwindling resources means difficult decisions have had to be made regarding reducing the sizes of faculty and reducing support services. This has the effect of adversely impacting the quality of education offered by institutions. The dynamics of the job market have also been impacted by tuition-free higher education with an influx of graduates who are saturating the job market in some fields, with the result being higher competition in some fields.

Sefoka (2018) posits that free higher education should be merit-based rather than free for all. This would ensure that the quality of higher education is not compromised and maintain academic excellence. The author argues that it is difficult for a developing nation such as South Africa to fund free education for all due to limited resources which are needed for a variety of critical needs. Hlaka and Sefoka (2022) point out that the South African government is not obligated by any legal instrument to offer free higher education for all. The authors further point to the lack of resources in South Africa as a significant obstacle to the delivery of free higher education.

De Jager and Baard (2019) found results which suggested free higher education in South Africa can lead to unsustainable demands on the fiscus. They point to South Africa lacking the key characteristics linked to countries that have been able to implement free higher education; these characteristics include a strong economy, a firm and wide tax base, low unemployment levels and limited access to higher education. The results of the study also point to the South African government needing to take care of more pressing demands such as those of basic education and health services thus reducing the portion of government resources that can be apportioned to free higher education. Other major issues highlighted in the study that hinder providing free education for all are corruption and wasteful expenditure by government departments. Dunga and Mncayi (2016) in a study conducted at a South African university found results suggesting that students did not believe the government should offer free education. The students felt it was not feasible while those who advocated for it felt it should be for the academically deserving only.

Mlambo, Hlongwa and Mubecua (2017) question the feasibility and sustainability of tuition-free education in South Africa. They point to the economic challenges faced by South Africa and the need to ensure that funds for free education are not supplanted by other departments. They offer several recommendations which can help the South African government maintain the current free higher education for low-income students regime. The first recommendation is that government should pursue private-public partnerships. With the private sector benefitting from graduates coming from Universities, it can participate in higher education through helping to fund students. The authors point to the need for new infrastructure in universities to accommodate the increase in students due to free education. The authors point to the need to increase funding in TVET colleges, TVET colleges provide vital education which is both practical and theoretical. The authors suggest that oversight over universities be increased to limit instances of wasteful expenditure and corruption.

The Public Service Association (2016) offered its perspective on South African higher education. The organisation pointed out that while the issue of fees had taken centre stage in debates about equity in higher education the focus should also be put on other structural matters. For instance, the organisation noted that one of the issues causing poor graduation rates in Universities was poor basic education thus there was a need for government to address the issue of basic education. The organisation felt free education for all would not be feasible given the competing demands on the government purse.

Universities South Africa (USAf) (2017) support a cost-sharing model between the state and the students, this is based on the idea that higher education is both a public good and a private good. The organisation points to a need for a mechanism designed for the poor and academically deserving but contends that tuition-free education is not feasible in South Africa. The main concerns of the organisation are the lack of clarity on how the government will fund free education in the long term and the apparent lack of capacity and competence shown in the past by the government through not honouring its commitment to avail subsidies to universities.

Motala, Vally, and Maharajh (2018) argue that tuition-free higher education on its own is not adequate to solve the societal problems in South Africa. The authors suggest that students should receive funding for the full cost of study including food, books, accommodation, travel and registration fees. They suggest that funds for tuition-free education can be raised through taxation part of that taxation being increasing the tax paid by the top 10% of income earners in South Africa. The top 10% of earners in South Africa earn 60 to 65% of the income in South Africa, thus the authors argue this presents a large pool of tax revenue. The authors further suggest that there should be clear timelines set for plans to implement tuition-free higher education.

### Lessons learnt and implications

Fifteen articles were reviewed to assess their position on the feasibility of tuition-free education is feasible in South Africa. Several lessons were gathered from the articles. Authors on the subject of tuition-free higher education make a distinction between tuition-free higher education for all and tuition-free education for the poor and academically deserving ( Cloete, 2015). This distinction is important in the context of South African higher education, as the latter seems to receive support from most stakeholders.

A key consideration in the discussion of the feasibility of tuition-free higher education in South Africa is resource availability. The South African government operates within the confines of a limited budget ( Wangenge-Ouma, 2008). Given the competing demands of the South African fiscus, there is an argument that the funding of tuition-free higher education results in the defunding or underfunding of other important government services such as healthcare and social security programs.

The calls for tuition-free higher education in South Africa are not novel calls on the African continent. Other countries tried tuition-free higher education policies post-independence. These policies were instated to reverse colonial inequalities and aggressively promote education ( Langa et al., 2017). These well-meaning attempts at tuition-free higher education resulted in increased inequalities between the poor and, political and business elites. This is a cautionary tale for South Africa that the promotion of tuition-free higher education can have the unintended consequences of creating or furthering inequalities between elites and the poor. Another possible result of the migration of elites to foreign universities and private institutions was the result in the African countries that attempted to institute tuition-free higher education policies.

It is important to keep in mind that the quality of higher education offered by South African institutions needs to be maintained or improved upon. It is therefore for tuition-free higher education proponents to carefully consider the effect of the policy on the quality of higher education ( Badat, 2011). In the event of access increasing while quality drops, this will possibly result in a brain drain with qualified and experienced lecturers and researchers leaving for better countries and will result in the rich choosing private institutions.

Countries with free higher education systems have certain characteristics that South Africa seems to lack at the current moment. These characteristics include a strong economy, a firm and wide tax base, low unemployment levels and limited access to higher education ( de Gayardon, 2019). These characteristics play a role in ensuring tuition-free higher education is sustainably funded in the long term. A strong economy ensures the government has adequate resources to meet its obligations. Low unemployment levels are essential to ensure the government has a wide tax base that provides revenue for tuition-free higher education. South Africa has high unemployment levels and a relatively small proportion of the population that pays taxes. When this is coupled with corruption and wasteful expenditure (De Jager and Baard,2019), the sustainability of tuition-free higher education in South Africa is not assured.

The government of South Africa should not forget to look at other structural issues that are essential for improving access and participation in higher education. One of the issues is the need for improving the basic education system which has been producing a significant portion of students that are ill-equipped to participate in higher education ( The Public Service Association, 2016). The result is that even if some of the students are granted access a significant portion struggles to complete studies on time and others fail to complete their studies. It is important that in considering offering tuition-free higher education for all the government consider other issues that hinder student success. These include access to books, housing and transportation. A possible source of resources for the government to fund tuition-free higher education for all is through increasing taxes on the top 10% of income earners in South Africa who earn a combined 60 to 60% of income ( Motala, Vally, and Maharajh, 2018). However, since the bulk of tax revenue already comes from this demography such a policy will simply serve to overburden an already-burdened taxpayer group

## Conclusion

The scoping review was designed to assess the feasibility tuition-free education in South Africa. The study sought to assess the feasibility of free higher education in South Africa. Results from the scoping review indicate that free higher education for all is not deemed feasible in South Africa. There is a view that the government has finite resources and funding free higher education will mean other more deserving services are neglected ( DHET, 2018). Funding free higher education for all is also viewed as a tax on the poor, as most students from middle and upper-income households get admitted for higher education studies ( Cloete 2015, Wangenge-Ouma & Cloete, 2008).

A moral argument is also offered, with the indication that in a highly unequal society such as South Africa offering free higher education to the rich is morally indefensible ( Cloete, 2015). Thus the a need for a fee regime that looks at the ability of the household to pay. Learning from the history and experiences of developing countries can also help in understanding the feasibility of free higher education for all in South Africa. Langa, Wangenge-Ouma, Jungblut and Cloete (2017) point to the failure of several African countries to sustain tuition-free higher education for all, with the result being the entrenchment of inequalities as political and business elites reaped the benefits at the expense of the poor.

The current GDP levels in South Africa are insufficient to support higher education and the government needs to double the percentage of GDP earmarked for higher education (Yende and Mthombeni,2023). The current tuition-free education policy in South Africa is already leading to an increased burden on universities affecting the quality of teaching and support services. Structural issues need to be addressed for tuition-free education for all to be successful in South Africa, for instance, the government need to ensure the quality of basic education is improved ( The Public Service Association, 2016). Poor basic education means the poor fail to meet admission requirements for higher education, thus the inequalities the government seek to address will persist. The South African government’s management of the NSFAS loan does not inspire confidence that it will be able to sustain tuition-free higher education in the long term. The concern is that poor management, corruption and wastage of resources will lead to failure of tuition-free higher education policy ( USAf, 2017).

It seems there is a consensus that free higher education for all is not feasible in South Africa. There is an understanding that free higher education for all and free education for the poor mean two different things. Thus some support free education for the poor as a moral imperative, given the high level of inequality in South Africa. In contrast to the majority of articles reviewed Motala et al. (2018) indicated support for free higher education and believe it is feasible if the government increase tax revenue through increasing tax revenues on the top 10% earners. However, there are concerns that increasing taxes will put a further burden on already overburdened taxpayers.

## Data Availability

No data are associated with this article. Name of repository- Zenodo
• Title- Exploring the Potential of tuition-free HE PRISMA-ScR-Fillable-Checklist 2024.docx • DOI- 10.5281/zenodo.11120736. • Full reference- Ramasu, T. K., & Kanakana - Katumba, M. G. (2024). Exploring the Potential of tuition-free HE PRISMA-ScR-Fillable-Checklist 2024.docx. Zenodo. https://doi.org/10.5281/zenodo.11120737. Data are available under the terms of the Creative Commons Attribution 4.0 International license (CC-BY 4.0).
