# Supplementary material for: Exploring the Potential for Tuition-Free Higher Education in South Africa: A Scoping Review
Source: F1000Res. 2024 Aug 29;13:526. Originally published 2024 May 22. [Version 2] doi: 10.12688/f1000research.150265.2 (PMC11459122; doi:10.12688/f1000research.150265.2)
Supplement: Peer review history

# Reviewer report: Reviewer response for version 2

**DOI:** 10.5256/f1000research.170339.r323308

**Published:** 2024-10-07

**Authors:** Isaiah Mmatipe Sefoka

**Affiliations:** 1 University of Limpopo, Limpopo Province, South Africa

The paper sought to assess the feasibility of a tuition-free higher education in South Africa. The assessment is informed by the recent large number of students pursuing higher education in South Africa and across. The choice of the scoping review was due to the need for an understanding of the current state of play in research on the feasibility of tuition-free higher education in the country. The paper suggest that tuition-free higher education for all is not feasible in South Africa owing to its lack of the necessary resources to finance tuition-free higher education for all. Although there are views to the effect that tuition-free higher education is feasible through increasing tax revenues, this paper concludes that tuition-free higher education is not feasible and having to increase taxes will obviously put a further burden on already overburdened taxpayers.    

 This to note, the paper is well written and is of good quality and standard. Moreover, the language adopted in the paper is good and acceptable. Generally the paper is well articulated and outlines contemporary and pertinent issues, provides thoughtful and robust discussion and offer good conclusion. Over and above, the paper provides well thought strategies, solutions or way forward to the problem identified.

 However, there are identified layout and grammatical errors of limited extent and other suggestions as indicated below which authors may consider attending to, in an attempt to perfect the paper.

 1. The words "thus the is" in line number 9 of the last paragraph of introduction sound like it could read "Thus there is ..."

 2. The word "as" between ... scheme loan and NSFAS on line 3 of paragraph 3 of the evolution of South African policies on higher education, make the sentence not readable. authors may consider revisiting same.

 3. Revisit the first sentence under "lessons learnt and implications".

 4. Put a full stop at the end of the last sentence of paragraph 6 under "lessons learnt and implications".

 5. The word "of" appears to be missing in line 1 under conclusion between the words "feasibility" and "tuition-free education in ..." 

 6. The words "Thus the a need" in line 2 of paragraph 2 under conclusion may read "thus there is a need" or thus a need for ...

Are the rationale for, and objectives of, the Systematic Review clearly stated?

Yes

Is the statistical analysis and its interpretation appropriate?

Yes

If this is a Living Systematic Review, is the ‘living’ method appropriate and is the search schedule clearly defined and justified? (‘Living Systematic Review’ or a variation of this term should be included in the title.)

Yes

Are sufficient details of the methods and analysis provided to allow replication by others?

Yes

Are the conclusions drawn adequately supported by the results presented in the review?

Yes

Reviewer Expertise:

Legal education, Labour law, Alternative Dispute Resolution (ADR) and Human Rights

I confirm that I have read this submission and believe that I have an appropriate level of expertise to confirm that it is of an acceptable scientific standard.

# Reviewer report: Reviewer response for version 2

**DOI:** 10.5256/f1000research.170339.r318622

**Published:** 2024-09-24

**Authors:** Zayd Waghid

**Affiliations:** 1 Faculty of Education, Cape Peninsula University of Technology, Cape Town, Western Cape, South Africa

I am satisfied with the revised article.

Are the rationale for, and objectives of, the Systematic Review clearly stated?

Partly

Is the statistical analysis and its interpretation appropriate?

No

If this is a Living Systematic Review, is the ‘living’ method appropriate and is the search schedule clearly defined and justified? (‘Living Systematic Review’ or a variation of this term should be included in the title.)

Not applicable

Are sufficient details of the methods and analysis provided to allow replication by others?

Yes

Are the conclusions drawn adequately supported by the results presented in the review?

No

Reviewer Expertise:

Social justice education

I confirm that I have read this submission and believe that I have an appropriate level of expertise to confirm that it is of an acceptable scientific standard.

# Reviewer report: Reviewer response for version 1

**DOI:** 10.5256/f1000research.164817.r291745

**Published:** 2024-07-17

**Authors:** Zayd Waghid

**Affiliations:** 1 Faculty of Education, Cape Peninsula University of Technology, Cape Town, Western Cape, South Africa

This is a very important study and one that has, since the implementation of the South African government's policy around "free higher education", warranted rigorous empirical research.  

 However, the article has several shortcomings. In this regard, the article is not suitable at this point for being indexed and would require major revisions.

 Firstly, there is the absence of a research question to assist with the direction and focus of the study. Although, the authors have included the aim of the study, which is generally much broader than a research question, particularly since aims outline the objectives or goals of one's research, a research question is needed from the onset.

 Second, the results section is very poor. There needs to be a detailed description of the findings in relation to an RQ. It would also have made sense to have themes related to each finding and then to build on these in the discussion section.

 Third, the first section of the discussion section is rather poor in that it appears to be written like a literature review. One would have expected deeper discussions pertaining to the findings, particularly regarding its implications for policy and practice.

 Fourth, the conclusion section needs to underscore the study's limitations and offer recommendations for further inquiry. 

 Other comments:

 As far as I know, Egypt and Saudi Arabia are still considered developing nations. Please check your reference. 

 The article would benefit from being language edited to ensure that it enhances the overall flow of ideas and arguments throughout the article.

Are the rationale for, and objectives of, the Systematic Review clearly stated?

Partly

Is the statistical analysis and its interpretation appropriate?

No

If this is a Living Systematic Review, is the ‘living’ method appropriate and is the search schedule clearly defined and justified? (‘Living Systematic Review’ or a variation of this term should be included in the title.)

Not applicable

Are sufficient details of the methods and analysis provided to allow replication by others?

Yes

Are the conclusions drawn adequately supported by the results presented in the review?

No

Reviewer Expertise:

Social justice education

I confirm that I have read this submission and believe that I have an appropriate level of expertise to state that I do not consider it to be of an acceptable scientific standard, for reasons outlined above.
